# Morphine Efficacy, Tolerance, and Hypersensitivity Are Altered After Modulation of SUR1 Subtype K_ATP_ Channel Activity in Mice

**DOI:** 10.3389/fnins.2019.01122

**Published:** 2019-10-22

**Authors:** Cole Fisher, Kayla Johnson, Travis Okerman, Taylor Jurgenson, Austin Nickell, Erin Salo, Madelyn Moore, Alexis Doucette, James Bjork, Amanda H. Klein

**Affiliations:** ^1^Department of Pharmacy Practice and Pharmaceutical Sciences, College of Pharmacy, University of Minnesota, Duluth, MN, United States; ^2^Department of Biomedical Sciences, Medical School Duluth, Duluth, MN, United States

**Keywords:** opioid, tolerance, withdrawal, K_ATP_ channels, SUR1, analgesia, antinociception

## Abstract

ATP-sensitive potassium (K_ATP_) channels are found in the nervous system and are downstream targets of opioid receptors. K_ATP_ channel activity can effect morphine efficacy and may beneficial for relieving chronic pain in the peripheral and central nervous system. Unfortunately, the K_ATP_ channels exists as a heterooctomers, and the exact subtypes responsible for the contribution to chronic pain and opioid signaling in either dorsal root ganglia (DRG) or the spinal cord are yet unknown. Chronic opioid exposure (15 mg/kg morphine, s.c., twice daily) over 5 days produces significant downregulation of Kir6.2 and SUR1 in the spinal cord and DRG of mice. *In vitro* studies also conclude potassium flux after K_ATP_ channel agonist stimulation is decreased in neuroblastoma cells treated with morphine for several days. Mice lacking the K_ATP_ channel SUR1 subunit have reduced opioid efficacy in mechanical paw withdrawal behavioral responses compared to wild-type and heterozygous littermates (5 and 15 mg/kg, s.c., morphine). Using either short hairpin RNA (shRNA) or SUR1 cre-lox strategies, downregulation of SUR1 subtype K_ATP_ channels in the spinal cord and DRG of mice potentiated the development of morphine tolerance and withdrawal. Opioid tolerance was attenuated with intraplantar injection of SUR1 agonists, such as diazoxide and NN-414 (100 μM, 10 μL) compared to vehicle treated animals. These studies are an important first step in determining the role of K_ATP_ channel subunits in antinociception, opioid signaling, and the development of opioid tolerance, and shed light on the potential translational ability of K_ATP_ channel targeting pharmaceuticals and their possible future clinical utilization. These data suggest that increasing neuronal K_ATP_ channel activity in the peripheral nervous system may be a viable option to alleviate opioid tolerance and withdrawal.

## Introduction

Potassium channels play a crucial role in controlling the excitability of neurons, including nociceptors and second order neurons in the spinal cord (Rasband et al., 2001). Many potassium channels are indirectly coupled to G-protein signaling pathways, including those from opioid receptors. These channels include G protein-activated inwardly rectifying potassium (GIRK, Kir3) and potassium channel subfamily K member 2 (TREK-1, KCNK2) channels (Marker et al., 2002; Devilliers et al., 2013). ATP-sensitive potassium (K_ATP_) channels are also involved in opioid signaling, and are expressed in nociceptors and spinal cord neurons (Ocaña et al., 1990; Rodrigues and Duarte, 2000; Stötzner et al., 2018). In the brain, chronic morphine exposure decreases the gating kinetics of potassium channels, indicating that decreased downstream opioid receptor coupling may account for opioid tolerance in the nervous system (Chen et al., 2000). The identity of these potassium channels is still up for debate, but it creates a compelling argument that activation of potassium channels could represent an interesting therapeutic solution to morphine tolerance and withdrawal due to their ability to hyperpolarize the membranes in the peripheral and central nervous system and make the neurons less excitable (Christie, 2008).

K_ATP_ channels are heterooctomers composed of either Kir6.2/SUR1, Kir6.2/SUR2, Kir6.1/SUR1, or Kir6.1/SUR2 subunits in the nervous system. K_ATP_ channels present a potentially attractive peripheral target for the treatment of pain due to the expression of K_ATP_ channel subunits, including Kir6.2/SUR1 in dorsal root ganglia (DRG) and Kir6.1/SUR2 and Kir6.1/SUR1 the superficial dorsal horn (Zoga et al., 2010; Wu et al., 2011). K_ATP_ channels are known to regulate excitability in a variety of neurons in the peripheral and central nervous system and many studies have implicated that K_ATP_ channel activity and/or expression are involved in chronic pain (Kawano et al., 2009a, c; Du et al., 2011; Xia et al., 2014; Zhu et al., 2015; Al-Karagholi et al., 2017). The prominent assumption is uninjured primary afferent neurons express K_ATP_ channels and conduct current which is eliminated during neuropathic or inflammatory pain states.

Opioids are one of the most common potent analgesics for the treatment of severe pain, including codeine, hydrocodone, oxycodone, and fentanyl. Although opioids, in general, are not recommended as a first-line treatment for neuropathic pain (Volkow and Koroshetz, 2017), they are still prescribed long-term in 6–32% of patients (Callinan et al., 2017; DiBonaventura et al., 2017; Hoffman et al., 2017). Given that the prevalence of neuropathic pain in the general population, it is possible there are many thousands of patients regularly taking opioid medications for neuropathy. Chronic administration of opioids often leads to the development of analgesic tolerance, leading to dose escalation, which decreases the viability of long-term clinical opioid use. Other issues arising from chronic opioid therapy is the likelihood of withdrawal after the patient ceases therapy, the potential for dependence, the abuse and/or misuse of opioids. Extensive research work has been done to explain the role of various neurotransmitters, channels, and receptor systems in the development of withdrawal syndrome (Cahill et al., 2016) in addition to other unwanted side effects including respiratory depression and abuse liability. Paradoxically, one of the hallmark features of opioid tolerance and withdrawal is the hyperexcitability of the nervous system, including the reversal of tonic inhibition of cAMP signaling and voltage-gated calcium channel function with repeated opioid administration. Reversal of this hyperexcitability of neurons in the spinal cord and DRG, via potassium channels, may improve tolerance and decrease symptoms of withdrawal after chronic opioid use.

In pharmacological studies, K_ATP_ channels are implicated in acute opioid analgesia (Welch and Dunlow, 1993), in addition to opioid tolerance and withdrawal after chronic morphine administration (Seth et al., 2010). However, the precise K_ATP_ channel subunits and location (e.g., spinal cord versus peripheral nervous system) involved during acute and chronic opioid administration remains to be clarified. This study investigated the roles of K_ATP_ channels using pharmacological tools to selectively activate or inhibit the SUR1 K_ATP_ channel subtype, and also utilized genetic strategies to delete or reduce activity of SUR1 K_ATP_ channel subunits in the nervous system. Our data indicate mice lacking the SUR1 subtype K_ATP_ channels have decreased analgesic responses to morphine. Genetic knockdown of SUR1 through AAV9-shRNA and AAV9-Cre recombinase strategies in SUR1 flox mice potentiated development of morphine tolerance and withdrawal, while pharmacological activation of the SUR1 K_ATP_ channel subtype attenuates tolerance and withdrawal in mice. These results give new insight into the mechanisms behind opioid tolerance and withdrawal and may aid in the development of new therapeutic strategies to combat the opioid epidemic.

## Materials and Methods

### Animals and Breeding

All experimental procedures involving animals were performed and approved in accordance with the University of Minnesota Institutional Animal Care and Use Committee guidelines. Adult C57Bl6 mice were obtained via Charles River (5–6 weeks old, 20–25 g, Raleigh, NC, United States). Breeding pairs of SUR1 global knock out mice (SUR1 KO) and SUR1 floxed mice (SUR1 flox) mice were obtained from the laboratory of Dr. Joseph Bryan at the Pacific Northwest Research Institute (Seattle, WA, United States) as used in previous studies (Seghers et al., 2000; Nakamura and Bryan, 2014) and kept on a C57Bl6N background. Behavioral experiments were performed on adult mice (>5 weeks age, 20–28 g). Verification of genotype was performed as previously described (Seghers et al., 2000; Nakamura and Bryan, 2014; Luu et al., 2019). Mice were euthanized by isoflurane anesthesia (5%) followed by decapitation at the end of the study.

### Spinal Nerve Ligation

To create a model of neuropathic pain in mice, spinal nerve ligation (SNL) was performed on adult male C57Bl6 mice (Kim and Chung, 1992; Ye et al., 2015). The SNL surgery was completed within 20 min as previously described on mice under isoflurane anesthesia (1–3%) in oxygen (Klein et al., 2018). Using aseptic technique, the lumbar spinal region was exposed. After removal of the sixth lumbar transverse process, the left fifth lumbar spinal nerve was tightly ligated with 6-0 silk suture and cut. Lumbar connective tissue, muscle and skin were closed by 4-0 re-absorbable sutures. The right spinal nerves were left intact as a control (contralateral). Mice were returned to their home cages post-operatively and, after 6 weeks, some SNL animals were treated chronically with morphine for 5 days (see below).

### Drugs and Delivery

Diazoxide, NN414, pinacidil, nicorandil, gliclazide or glyburide (Sigma Aldrich, St. Louis, MO, United States or Tocris, Minneapolis, MN, United States) were initially diluted in DMSO, then further diluted to a final concentration of 100 μM in 5% DMSO in saline (vehicle). Compounds in this study were administered at concentrations found in previous behavioral studies (Rodrigues and Duarte, 2000; Alves et al., 2004a, b; Maia et al., 2006; Ortiz et al., 2010). Mice were gently restrained during injections using a Plexiglas restrainer. Intraplantar injections were performed by inserting a 27–33 ga needle into the paw at an angle, with the bevel facing the skin, while 10 μL of solution was delivered over 1 min (Klein et al., 2011, 2015). Subcutaneous injections (100 μL) were delivered by grasping the neck skin and pulling upward to create a tent. Typically, mice were tested with different chemicals; to avoid carryover effects, a minimum of 7 days elapsed between successive tests.

Morphine efficacy was determined by using an escalating dose response curve (5–20 mg/kg, s.c.) as described previously in other studies (de Guglielmo et al., 2014; Klein et al., 2018). Morphine doses were calculated to be similar to the human morphine equivalent doses (Nair and Jacob, 2016) found in neuropathic pain and other chronic pain patients (Dunn et al., 2010; Moulin et al., 2015; Cooper et al., 2017). For morphine tolerance experiments in mice, baseline mechanical or thermal paw withdrawal testing was performed before administration of, morphine (Sigma Chemical, St. Louis, MO, United States) subcutaneously (15 mg/kg twice per day, s.c., ∼0800 and ∼1800 h, 100 μL) on days 1–5 as done in previous studies (Liang et al., 2011, 2013; de Guglielmo et al., 2014). Threshold testing was performed ∼30 min post morphine administration in the morning. On day 6, to determine the degree of opioid withdrawal in the absence of morphine, mice paw withdrawal thresholds were assessed ∼18 h after the final dose of morphine.

### Mechanical Paw Withdrawal Thresholds

Mice were previously acclimated to the testing environment, a mesh floor with individual clear acrylic chambers. Mechanical paw withdrawal thresholds were determined by use of electronic von Frey testing equipment (Electric von Frey Anesthesiometer, 2390, Almemo^®^ 2450, IITC Life Science, Woodland Hills, CA, United States) (Martinov et al., 2013). The plantar surface of the hind paws were gently pressed with the probe until the force elicited a nocifensive response (i.e., paw lifting, jumping, etc.). Baseline measurements were collected five times from both the right and left hind paw with an interstimulus interval of 1 min.

### Thermal Radiant Heat Withdrawal Latency

Mice were acclimated to the testing environment, a glass floor heated to 30°C with individual clear acrylic chambers, on at least 3 separate days. The modified Hargreaves method was used to measure thermal paw withdrawal latency (Plantar Test Analgesia Meter, 400, IITC, Woodland Hills, CA, United States) (Cheah et al., 2017). Paw withdrawal latencies were determined by the amount of time a heat radiant beam of light focused on the plantar surface of the hind paw was required to elicit a nocifensive response (e.g., paw lifting, tapping, shaking, jumping or licking the paw). A maximum time limit of 20 s exposure to the beam was observed to avoid tissue damage. Baseline measurements were collected three times from both the right and left hind paw with an interstimulus interval of at least 2 min.

### Adeno-Associated Virus Serotype 9 (AAV9)-Mediated SUR1 Knockdown

Effective and ongoing knockdown of SUR1 K_ATP_ channel subunits in the spinal cord and dorsal root ganglia was achieved by the delivery of AAV vectors via intrathecal injection. Two different AAV9 viral strategies were used in this study, short hairpin (shRNA) gene knockdown in C57Bl6 mice (Hirai et al., 2012) and AAV9-mediated Cre expression in SUR1 flox mice. Gene knockdown of *Abcc8* (protein product: SUR1) using shRNA was achieved using AAV9-GFP-U6-m-*ABCC8*-shRNA and AAV9-GFP-U6-scramble-shRNA (shAAV-251792 and 7045, titer: 10^13 GC/mL, in PBS with 5% glycerol, Vector Biolabs, Malvern, PA, United States). AAV9-hSyn-GFP-Cre and AAV9-hSyn-GFP-ΔCre (10 μL containing ∼10^13^ vector genomes, University of Minnesota Viral Vector Core, Minneapolis, MN, United States) were delivered by direct lumbar puncture in awake mice as previously described (Vulchanova et al., 2010; Schuster et al., 2013; Pflepsen et al., 2019) and behavioral assessments were performed 1–8 weeks post injection. Verification of mRNA knockdown was achieved using quantitative polymerase chain reaction (qPCR) of harvested lumber spinal cords and DRG (see section ‘Quantitative PCR’). Histological sections were taken from some animals in order to demonstrate successful delivery of AAV vectors by visualization of green fluorescent protein (GFP). Sections of spinal cord and DRG (10 μM, Leica CM3050) were mounted onto electrostatically charged slides and images were collected using a Nikon TiS Microscope and associated software.

### Cell Culture

Human neuroblastoma SH-SY5Y cells (CRL-2266, ATCC^®^, Manassas, VA, United States) were maintained in Dulbecco’s Modified Eagle’s Medium/Hams F-12 with L-glutamine (10-090-CV, Corning^®^, Corning, NY, United States) supplemented with 10% v/v fetal bovine serum and kept within a humidified incubator at 37°C with 5% CO_2_. Cells were sub-cultured when confluency reached 70–80% and reseeded at a 1:5 ratio. SH-SY5Y cells were plated at 7.5 × 10^4^ cells per well in a clear bottom, black-walled 96-well plate coated with poly-D-lysine (354210, Corning^®^, Corning, NY, United States) and incubated for 3 days or until 90% confluent in complete media or in complete media with 10 μM morphine.

DRG were extracted from SUR1 WT and KO mice (1–7 months, 20–40 g) and placed into Petri dishes containing ice cold 1X HBSS. Ganglia were minced and incubated in 32 μL papain (27.3 U/mg, no. 3126; Worthington Biochemical, Lakewood, NJ, United States) with 1 mg l-cysteine (Sigma) in 1.5 mL 1X HBSS for 10 min in a 37°C rocking water bath. Cells were centrifuged at 1600 rpm for 2 min, the supernatant was aspirated, and the DRG were incubated in 2 mg/mL collagenase type II (CLS2; Worthington Biochemical) in 1X HBSS for 10 min in a 37°C rocking water bath. Cells were centrifuged at 1600 rpm for 2 min and gently triturated using fire-polished glass pipettes with 2 mL pre-warmed complete media consisting of Eagle’s Minimum Essential Medium with Earle’s salts and L-glutamine (10-010-CV, Corning^®^, Corning, NY, United States) supplemented with 10% v/v horse serum, 1% v/v 100X MEM vitamin solution and 1% v/v penicillin-streptomycin (Gibco, Thermo Fisher Scientific, Waltham, MA, United States). DRG were centrifuged 2 min at 1000 rpm, the media was aspirated, and the pellet was resuspended in 5 mL pre-warmed complete media. Isolated DRG were plated in 100 μL aliquots into clear bottom, black-walled 96-well plates coated with poly-D-lysine and kept within a humidified incubator at 37°C with 5% CO_2_. Fluorescence intensity plate reading (FLIPR) was performed on cells 24 h post isolation.

### Rotarod Performance Test

Agility assessments were conducted with the Rotamex-5 automated rotarod system (0254-2002L, 3 cm rod, Columbus Instruments, Columbus, OH, United States). Mice were placed onto a stationary knurled PCV rod suspended in the air. The rotation speed of the rod was initially set to 4 rpm and gradually increased in 1 rpm increments at 30-s intervals until animals fell off the rod or reached a speed of 14 rpm (300 s) (Deacon, 2013). At least two tests per animal were administered to ensure an animal did not fall off the rod prematurely.

### Burrowing Test

Mice were acclimated to burrowing tubes for ∼2 h at least 1 day before formal testing. The burrows were made from plastic pipe with a 6 cm diameter and 5 cm machine screws were used to elevate the bottom of the tube 3 cm from the floor (Deacon, 2012). During testing, each mouse was placed in an individual cagewith a burrowing tube containing 500 g of pea gravel. The amount of gravel remaining in the tube after 2 h was used to calculate the total percent of gravel displaced from the burrow.

### FLIPR Potassium Dye Assay

Potassium channel activity of cells was measured using FLIPR Potassium Assay Kit (Molecular Devices, LLC, San Jose, CA, United States) according to manufacturer instructions (Shieh et al., 2007). Briefly, cell culture media was replaced with a 1:1 mixture of 1X Hank’s Balanced Salt solution with 20 mM HEPES, and FLIPR Loading Dye with 5 mM probenecid. Cells were incubated for 1 h at room temperature in the dark. Drugs were added to each well (in 10 μL, 15% DMSO + 42.5% ethanol) and incubated 10 min. Fluorescence data was collected after the addition of 48 μL of a 10 mM solution of thallium sulfate per well. Background fluorescence was measured for 30 s at a 21 s interval and fluorescence post thallium addition was monitored for 600 s with a 21 s interval using a BioTek Synergy 2 (BioTek, Winooski, VT, United States) multi-well plate reader equipped with excitation filter of 485/20 nM and emission filter 528/20 nM. Fluorescence readings were converted to total fluorescence over vehicle by subtracting the vehicle reading at each time point from all treatments summed over the entire period.

### MTT Assay

To assess overall cellular health after morphine treatment SH-SY5Y cells were plated at 7.5 × 10^4^ cells per well in a clear 96-well plate coated with poly-D-lysine (354210, Corning^®^, Corning, NY, United States) for 3 days in complete media or in complete media with 5–1,500 μM morphine in a humidified incubator at 37°C with 5% CO_2_. After 3 days, media was removed and replaced with 100 μL pre-warmed complete media along with 10 μL of a 5 mg/mL thiazolyl blue tetrazolium bromide (00697, Chem-Impex International, Inc., Wood Dale, IN, United States) solution in 1X phosphate buffered saline and incubated 4 h (van Meerloo et al., 2011). Next, 100 μL of a 0.01 N HCl solution with 10% w/v sodium dodecyl sulfate was added. Plates were wrapped in parafilm and incubated overnight. Absorbance was read at 570 nm using a BioTek Synergy 2 multi-well plate reader.

### Quantitative PCR

RNA samples (50 ng) were collected from mouse tissues using the RNeasy Mini Kit (Qiagen, Germantown, MD, United States) with DNAse digestion steps. Complimentary DNA synthesis was performed using the Omniscript RT Kit and protocol from Qiagen with primers used previously (Luu et al., 2019). Quantitative PCR was performed with SYBR Green I dye using LightCycler 480 technology (Roche, Branchburg, NJ, United States). The cDNA copy number was typically quantified against a ≥5 point, 10-fold serial dilution of a gene specific cDNA standard. Internal controls included negative RT-PCR samples. Comparative expression versus a housekeeping gene, *18S* were used to create normalized data which were used for statistical analysis against saline treated animals. Fold expression of each gene of interest was presented by the ratio of: (mean concentration)/(mean concentration of *18S*) compared to saline-treated controls.

SH-SY5Y cells were collected from 12-well plates via trypsinization, pelleted, flash frozen in liquid nitrogen and stored at −80°C. One μg of total mRNA was used for cDNA synthesis using the Omniscript RT Kit and protocol from Qiagen. Quantitative PCR was performed with SYBR Green I dye using LightCycler 480 technology as above. Primers used for SH-SY5Y cells were as follows: *18S*: forward 5′-TCAACTTTCGATGGTAGTCGCCGT-3′ reverse 5′-TCCTTGGATGTGGTA GCCGTTTCT-3′. *ABCC8*: forward 5′-GACCAGGGGGTCCTCAACAA-3′ reverse 5′-ATGTG CAC CTTGGAGCTCTG-3′. *ABCC9*: forward 5′-ATCCTCGGTGAG ATGCAGAC-3′ reverse 5′-CTGTTCCTACTTCTGGTTGCT-3′. *KCNJ8*: forward 5′-GGCTGCTCTTCGCTATCATGT-3′ reverse 5′-CTCCCTCCAAACCCAA TGGTA-3′. *KCNJ11*: forward 5′-A AGAAGTGAAG TGGGACCCAGG-3′ reverse 5′-GCTGGCCT CACTTCTGAGATA-3′. *OPRM1*: forward 5′-CA TCACGATCA TGGCCCT-3′ reverse 5′-TCTGCCAGAGCAAGGTTGAA-3′.

### C-Fiber Compound Action Potentials

Compound action potentials (CAPs) were measured from the sciatic nerve of SUR1 flox mice 8 weeks after intrathecal injection of either AAV9-hSyn-GFP-Cre or AAV9-hSyn-GFP-ΔCre. Sciatic nerves were dissected from the hind limbs of mice and recordings were performed the day of harvesting. One nerve from each mouse was mounted in a chamber filled with superficial interstitial fluid. Electrical stimulation was performed at a frequency of 0.3 Hz with electric pulses of 100-μs duration at 100–10,000 μA delivered by a pulse stimulator (2100, AM Systems, Carlsborg, WA, United States). Evoked CAPs were recorded with electrodes placed 10 mm from the stimulating electrodes. Dapsys software was used for data capture and analysis (Brian Turnquist, Bethel University, St. Paul, MN, United States)^1^. The stimulus with the lowest voltage producing a detectable response in the nerve was determined the threshold stimulus. The stimulus voltage where the amplitude of the response no longer increased was determined to be the peak amplitude. The conduction velocity was calculated by dividing the latency period, the time from stimulus application to neuronal initial response, by the stimulus-to-recording electrode distance.

### Data Analysis

Appropriate *t*-test or one-way, two-way or repeated measures ANOVA followed by *post hoc* analysis were performed to determine the significance of treatment groups for gene expression, FLIPR data, mechanical and thermal paw withdrawals, and CAP single-unit recordings. Gene expression of K_ATP_ channel subunits and pharmacological behavioral data were compared to a saline treated control group and a Dunnett’s *post hoc* test was used to analyze this data. FLIPR and behavioral tests using SUR1 WT, Het, or KO mice were compared to each other to determine if partial loss of SUR1 function would contribute to differences seen in potassium flux or antinociception, respectively, therefore a Tukey *post hoc* test was used for these data. All statistical analyses were carried out with Prism 6.0 (GraphPad Software Inc., San Diego, CA, United States) or SPSS (Statistics 24, IBM, Chicago, IL, United States). The data are presented as means ± standard error, 95% confidence intervals (CI), and *p* < 0.05 was considered statistically significant.

## Results

### Alterations of KATP Channel Expression in Spinal Cord and Dorsal Root Ganglia in Morphine Tolerant Mice After Nerve Injury

**FIGURE 1:**
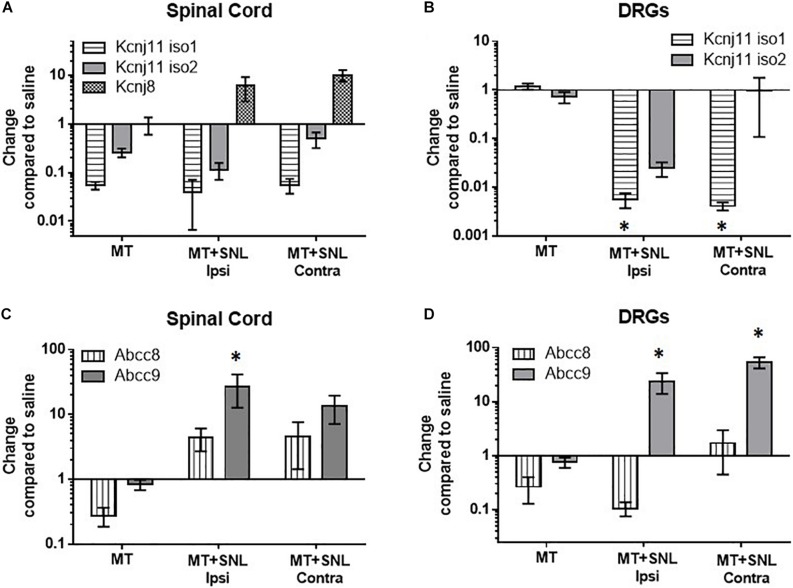
Decreased mRNA expression of *Kcnj11* and increased SUR2 expression in morphine tolerant mice. Morphine tolerant (MT) mice were treated twice daily with 15 mg/kg (s.c.) morphine for 5 days. Gene expression of (MT) and morphine tolerant mice after spinal nerve ligation (MT + SNL) are compared to saline treated mice as a control. The spinal cords and dorsal root ganglia (DRG) of MT + SNL animals were compared on the ipsilateral, injured side (i.e., ipsi) as well as the contralateral, non-injured side (i.e., contra). **(A)** The expression of Kcnj subunits were not significantly altered in the spinal cord (*n* = 5–6/group). *Kcnj11* iso1 potassium channel subunits were significantly decreased in the **(B)** dorsal root ganglia (DRG) of MT + SNL mice [two-way ANOVA, Dunnett’s *post hoc* compared to saline, *F*(3,52) = 3.8, *p* = 0.015, CI_MT+SNL Ipsi_ = 0.1382 to 2.126 and CI_MT+SNL Contra_ = 0.1367–2.127, *n* = 6–9/group]. **(C)** *Abcc9* subunit expression was significantly increased in the spinal cord compared to saline treated tissues [two-way ANOVA, Dunnett’s *post hoc* compared to saline, *F*(3,50) = 5.86, *p* = 0.0017; CI_MT+SNL Ipsi_ = −40.26 to −11.66, *n* = 5–9/group]. **(D)** *Abcc9* subunit expression was also significantly increased in the DRG of MT + SNL animals [two-way ANOVA, Dunnett’s *post hoc* compared to saline, *F*(3,52) = 14.98, *p* < 0.001, CI_MT+SNL Ipsi_ = −38.37 to −7.133 and CI_MT+SNL Contra_ = −68.38 to −37.15, *n* = 6–9/group]. Asterisk indicates statistical significance (*p* < 0.05).

To determine if chronic morphine exposure affects expression of K_ATP_ channels, we used qPCR to measure mRNA copies of pore-forming (Kir6.1 and Kir6.2) and regulatory (SUR1 and SUR2) K_ATP_ channel subunits. Morphine tolerant (MT) mice were treated twice daily with 15 mg/kg (s.c.) of morphine for 5 days. Gene expression of MT and morphine tolerant mice after spinal nerve ligation (MT + SNL) were compared to the same control group mice treated with saline for the same period of time (100 μL saline, sc) and plotted as change compared to saline to access expression changes across all groups. The spinal cords and DRG of MT + SNL animals were separated into the ipsilateral, injured side (i.e., ipsi) and contralateral, non-injured side (i.e., contra). The expression of the pore-forming Kcnj subunits were not significantly altered in the spinal cord compared to control animals (Figure 1A). *Kcnj11* iso1 potassium channel subunits were significantly decreased in the DRG of MT + SNL mice [Figure 1B, two-way ANOVA, Dunnett *post hoc*, *F*(3,52) = 0.015, CI _MT+SNL Ipsi_ = 0.1382 to 2.126 and CI _MT+SNL Contra_ = 0.1367 to 2.127]. The expression of the regulatory *Abcc9* subunits were increased in the spinal cord from MT + SNL mice compared to saline treated mice [Figure 1C, two-way ANOVA, Dunnett *post hoc*, *F*(3,50) = 5.9, *p* = 0.0017; CI _MT+SNL Ipsi_ = −40.26 to −11.66]. Similarly, *Abcc9* subunit expression was also significantly increased in the DRG of MT + SNL mice [Figure 1D, two-way ANOVA, Dunnett *post hoc*, *F*(3,52) = 14.9, *p* < 0.001, CI_MT+SNL Ipsi_ = −38.37 to −7.133 and CI_MT+SNL Contra_ = −68.38 to −37.15]. Interestingly, the expression of *Abcc8* was increased in the spinal cord, but decreased in the DRG of MT + SNL mice. The comparisons of *Abcc8* against vehicle treated tissues were not statistically significant.

### Potassium Flux of Cultured Cells and DRG Are Decreased After Chronic Opioid Administration

**FIGURE 2:**
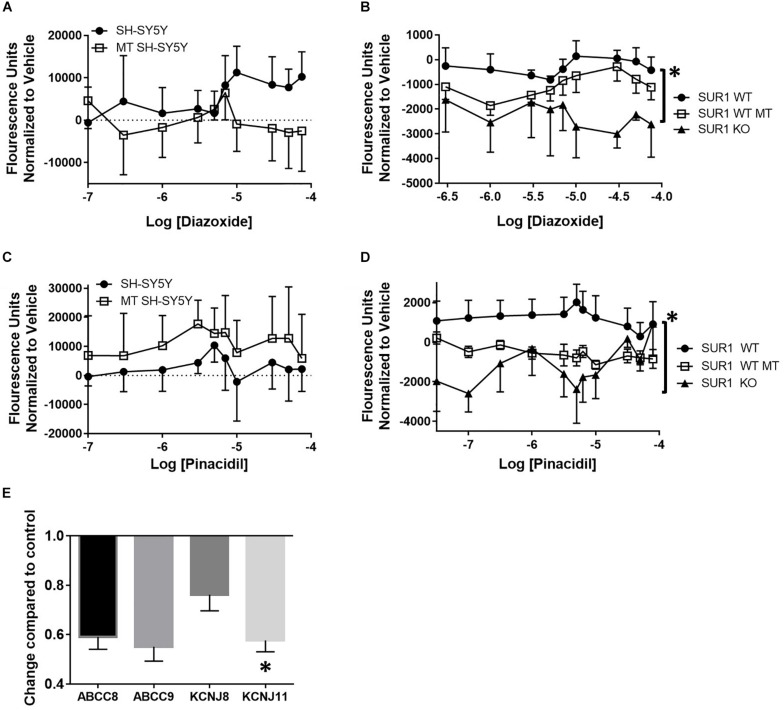
Decreased potassium flux of cultured cells and DRG after chronic opioid administration. **(A)** Increasing concentrations of a SUR1 agonist, diazoxide, result in increased fluorescence during potassium FLIPR in SH-SY5Y cells. SH-SY5Y cells previously incubated with 10 μM morphine for 72 h (morphine treated, MT, *n* = 7) have decreased potassium flux compared to non-morphine treated cells (SH-SY5Y, *n* = 7). **(B)** Potassium flux from DRG harvested from SUR1 WT morphine treated mice (15 mg/kg, s.c., 5 days) are slightly decreased compared to non-morphine SUR1 WT treated mice. DRG collected from SUR1 KO mice have a significantly attenuated potassium flux as a response to increasing concentrations of diazoxide compared to SUR1 WT DRG [*F*(2,12) = 9.902, *p* = 0.003, CI_SUR1 WT–SUR1 KO_ = 776.8–3110]. **(C)** Fluorescence values after increasing concentrations of a SUR2 agonist, pinacidil, are minimally increased during potassium FLIPR in SH-SY5Y cells. SH-SY5Y cells previously incubated with 10 μM morphine for 72 h (morphine treated, MT, *n* = 4) have decreased potassium flux compared to non-morphine treated cells (SH-SY5Y, *n* = 4). **(D)** Potassium flux from DRG harvested from SUR1 WT morphine treated mice (15 mg/kg, s.c., 5 days, *n* = 6) are slightly decreased compared to non-morphine SUR1 WT treated mice [*n* = 6, *F*(1, 10) = 4.735, *p* = 0.055]. DRG collected from SUR1 KO mice (*n* = 3) do not have any appreciable potassium flux as a response to increasing concentrations of pinacidil compared to SUR1 WT DRG. **(E)** K_ATP_ channel subunit expression is decreased after chronic administration of morphine (10 μM) in SH-SY5Y cells. Expression of KCNJ11 is significantly decreased in morphine treated compared to control SH-SY5Y cells (paired *t*-test, *p* = 0.045, *n* = 4). Asterisk indicates statistical significance (*p* < 0.05).

FLIPR was performed to investigate the changes in potassium channel flux in neurons occurring after chronic opioid administration. Potassium flux was measured in cultured neuroblastoma cells having a transcriptome similar to sensory neurons and DRG cultured from mice (Yin et al., 2016). Concentration dose-response curves were performed using K_ATP_ channel agonists targeting the SUR1-subtype, diazoxide, and the SUR2-subtype, pinacidil. Increasing concentrations of a SUR1 agonist, diazoxide, result in increased fluorescence during potassium FLIPR in SH-SY5Y cells. Morphine treated SH-SY5Y cells, previously incubated with 10 μM morphine for 72 h, had a decreased potassium flux compared to non-morphine treated cells (Figure 2A, SH-SY5Y, *n* = 7). Potassium flux from DRG harvested from SUR1 wild type (WT) morphine treated mice (SUR1 WT MT, 5 days, 15 mg/kg, s.c., 5 days) were slightly decreased compared to non-morphine treated SUR1 WT mice. DRG collected from SUR1 knock out (KO) mice had a significantly attenuated potassium flux as a response to increasing concentrations of diazoxide compared to SUR1 WT DRG [Figure 2B, repeated measures ANOVA, Tukey *post hoc*, *F*(2,12) = 9.9, *p* = 0.003, CI_SUR1 WT–SUR1KO_ = 776.8 to 3110]. This indicates SUR1 channel function was slightly minimized after morphine treatment. Since SUR1 KO DRG do not respond to diazoxide, this indicates that diazoxide is a valid agonist for studying potassium flux for SUR1-subtype K_ATP_ channels.

Increasing concentrations of a SUR2 agonist, pinacidil, in SH-SY5Y cells minimally increased potassium flux. Interestingly, SH-SY5Y cells previously incubated with 10 μM morphine for 72 h (morphine treated, MT, *n* = 4) had zero potassium flux compared to non-morphine treated cells (Figure 2C, SH-SY5Y, *n* = 4). Potassium flux after pinacidil exposure from DRG harvested from SUR1 WT morphine treated mice (SUR1 WT MT, *n* = 6) were decreased compared to non-morphine SUR1 WT treated mice, although this effect was not statistically significant [SUR1 WT, Figure 2D, *n* = 6, repeated measures ANOVA, Tukey *post hoc*, *F*(1,10) = 4.7, *p* = 0.055]. DRG collected from SUR1 KO mice (*n* = 3) had zero potassium flux as a response to increasing concentrations of pinacidil compared to DRG taken from SUR1 WT mice, indicating the function of SUR2 is also compromised in SH-SY5Y and mouse DRG chronically treated with morphine.

After chronic administration of morphine (10 μM, 72 h), SH-SY5Y cells have less K_ATP_ channel subunit expression compared to cells not treated with morphine. The expression of KCNJ11 was significantly decreased compared to control SH-SY5Y cells (Figure 2E, unpaired *t*-test, *p* = 0.045, *n* = 4). The decreased expression of K_ATP_ channel subunits after 10 μM opioid treatment was not due to morphine cell toxicity, as a MTT assay only indicated a significant retardation of proliferation in cells treated with >50 μM morphine compared to un-treated cells [ANOVA, Dunnett *post hoc*, *F*(13,70) = 377.8, *p* < 0.001, CI_10 μM_ = −0.03849 to 0.09082].

### Mice Deficient in SUR1-Type K_ATP_ Channels Have Attenuated Morphine Antinociception

**FIGURE 3:**
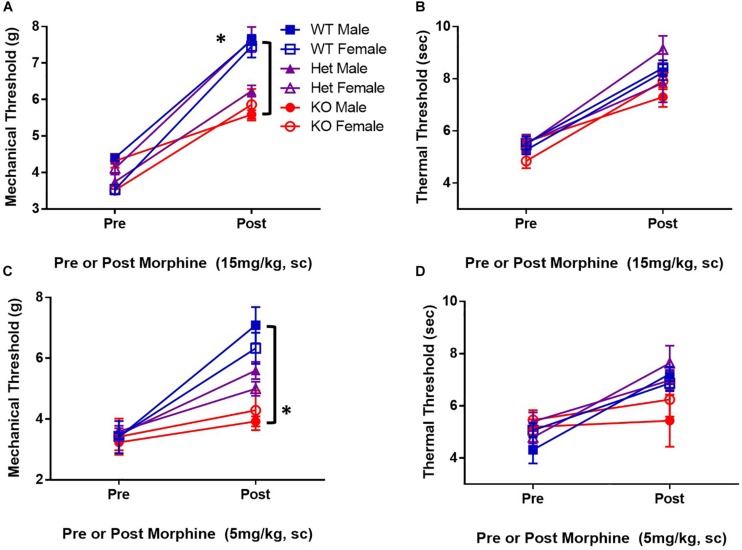
Mice deficient in the SUR1-subtype K_ATP_ channels have attenuated morphine antinociception. Mechanical and thermal thresholds were tested 30 min after morphine administration (15 mg/kg, sc, *n* = 5 mice/group) in SUR1 WT, SUR1 Het, and SUR1 KO male and female mice. **(A)** Significant differences were found comparing mechanical thresholds post 15 mg/kg morphine between SUR1 WT and Het vs. KO mice [repeated measures ANOVA, *F*(2,24) = 16.8, *p* < 0.001, CI_SUR1  WT  and  SUR1  KO_ = 1.34 to 0.52, CI CI_SUR1  HET  and  SUR1  KO_ = 1.02 to 0.21]. **(B)** No significant differences were found comparing thermal latency thresholds post 15 mg/kg morphine with regards to genotype. **(C)** Significant differences were found comparing mechanical thresholds post 5 mg/kg (s.c.) morphine between SUR1 WT and KO mice [repeated measures ANOVA, *F*(2,24) = 7.1, *p* = 0.004]. SUR1 KO mice had significantly lower mechanical thresholds compared to WT mice after morphine administration (CI = 2.24 to 0.46). **(D)** No significant differences were found comparing thermal latency thresholds post 5 mg/kg morphine with regards to genotype. No significant differences were found with regards to sex for either dose of morphine for either behavioral test. Asterisk indicates statistical significance (*p* < 0.05).

K_ATP_ channels are proposed downstream targets of opioid receptor signaling. To determine if K_ATP_ channels are sufficient for antinociception after μ-opioid receptor stimulation, the analgesic efficacy of morphine in mice lacking the SUR1 regulatory subunit of K_ATP_ channels was tested. The SUR1-subtype was chosen because it is found in the spinal cord and dorsal root ganglia, and altered SUR1 activity/expression has been implicated in chronic pain (Kawano et al., 2009b, c; Zoga et al., 2010) and our gene expression and potassium flux data (Figures 1, 2). Mechanical and thermal thresholds were tested in SUR1 wild type (WT), SUR1 heterozygous (Het), and SUR1 knockout (KO) male and female mice between 6 and 12 weeks of age, 30 min after morphine administration (15 mg/kg, s.c, *n* = 5 mice/group). SUR1 KO mice had attenuated morphine antinociception [Figure 3A, repeated measures ANOVA, Tukey *post hoc*, *F*(2,24) = 18.6, *p* < 0.001, CI_WT–KO_ = −1.34 to −0.52, CI_Het–KO_ = −1.02 to −0.21]. The loss of SUR1 did not affect thermal latency thresholds post 15 mg/kg morphine (Figure 3B). The loss of SUR1 is clearly important for mechanical antinociception post-morphine administration, but this was not the case for thermal antinociception. Similar results were also obtained for a lower dose of morphine, including a loss of mechanical antinociception [Figure 3C, repeated measures ANOVA, Tukey *post hoc*, *F*(2,24) = 7.1, *p* < 0.001, CI_WT–KO_ = −3.72 to −1.48] but not thermal antinociception (Figure 3D). No significant differences were found with regards to sex for either dose of morphine for either behavioral test.

### Knockdown of SUR1-Subtype KATP Channels in Mice Increases Peak Amplitude of Sciatic Nerve Electrical Conduction and Enhances Mechanical Hypersensitivity

**FIGURE 4:**
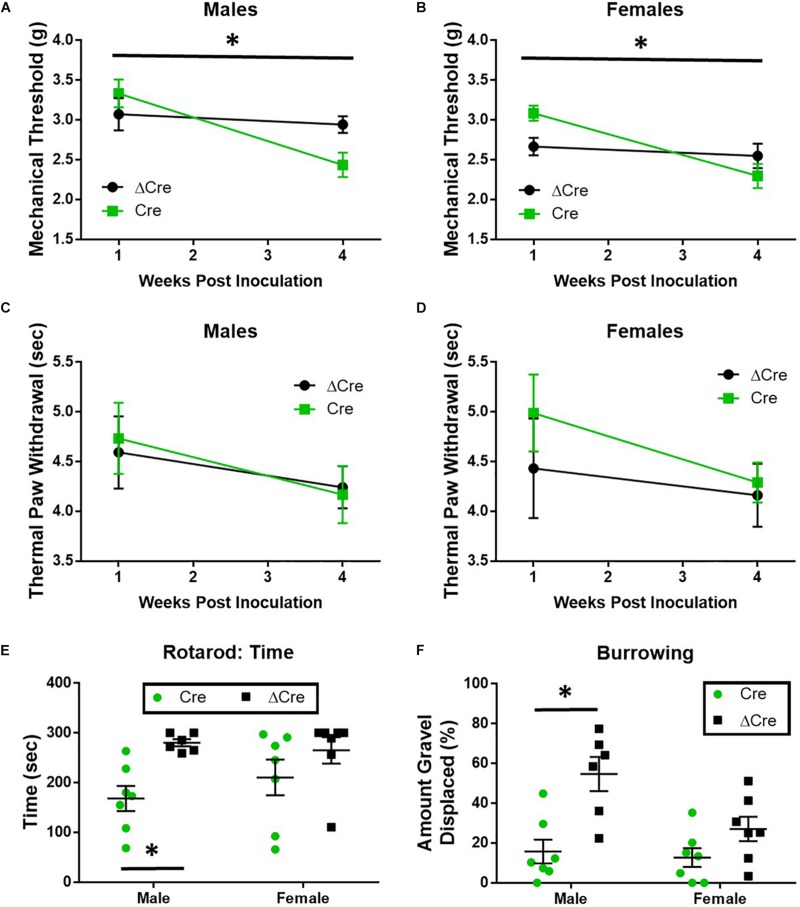
Mechanical paw withdrawal thresholds, burrowing, and rotarod performance are decreased in mice with conditional deletion of SUR1. **(A)** Mechanical paw withdrawal thresholds were significantly decreased across time in AAV9-hSyn-Cre male mice compared to AAV9- hSyn-ΔCre mice [*F*(1,22) = 5.23, *p* = 0.032]. **(B)** Mechanical paw withdrawal thresholds were significantly decreased across time in AAV9-hSyn-Cre female mice compared to AAV9- hSyn-ΔCre mice [*F*(1,25) = 8.43, *p* = 0.008]. There was no change in thermal thresholds between viral treatments in male **(C)** or female **(D)** mice. **(E)** The change in time spent on a rotating rod was significantly lower in AAV9-hSyn-Cre male mice compared to AAV9- hSyn-ΔCre mice [two-way ANOVA, *F*(1,23) = 9.64, *p* = 0.005]. **(F)** The amount of burrowing was significantly lower in AAV9-hSyn-Cre male mice compared to AAV9- hSyn-ΔCre mice [two-way ANOVA, *F*(1,23) = 17.5, *p* = 0.0004] and a significant sex effect was also found [*F*(1,23) = 5.81, *p* = 0.024]. Asterisk indicates statistical significance (*p* < 0.05).

The loss of SUR1 from the nervous system may ultimately contribute to chronic pain and increase the development of morphine tolerance and withdrawal through increased excitability of nociceptors. In order to restrict the downregulation of K_ATP_ channels to the lumbar spinal cord and DRG, instead of a global loss throughout the nervous system, two different AAV9 strategies were employed. For the first strategy, an AAV9-shRNA virus was introduced via intrathecal injection into male adult C57Bl6 mice (Luu et al., 2019). AAV9-shRNA-Abcc8 has been shown to significantly decrease mRNA expression of *Abcc8* in the lumbar spinal cord, DRG, and sciatic nerves of inoculated mice (Luu et al., 2019). The second strategy, an AAV9-Cre virus was administered by intrathecal injection into SUR1 flox mice and morphine dose response curves were performed 4 weeks later. Mechanical hypersensitivity was previously found in global SUR1 KO mice and *Abcc8* shRNA treated mice (Luu et al., 2019). SUR1 flox mice intrathecally inoculated with AAV9-hSyn-Cre also had significantly decreased mechanical paw withdrawal thresholds in male [Figure 4A, repeated measures ANOVA, *F*(1,22) = 5.23, *p* = 0.032] and female mice [Figure 4B, repeated measures ANOVA, *F*(1,25) = 8.43, *p* = 0.008]. Thermal paw withdrawal thresholds were not significantly altered (Figures 4C,D). Interestingly, we did find other behaviors that were altered after SUR1 conditional knockdown. AAV9-hSyn-Cre virus treated animals displayed decreased burrowing and rotarod activity in mice following conditional deletion of SUR1. The change in time spent on a rotating rod was significantly different between virus treatment [Figure 4E, two-way ANOVA, *F*(1,23) = 9.6, *p* = 0.005] and the amount of burrowing was significantly different between virus treatment groups [Figure 4F, two-way ANOVA, *F*(1,23) = 17.5, *p* = 0.0004]. A significant sex effect was also found with regards to burrowing behavior [Figure 4F, *F*(1,23) = 5.8, *p* = 0.024] but not with rotarod testing.

**FIGURE 5:**
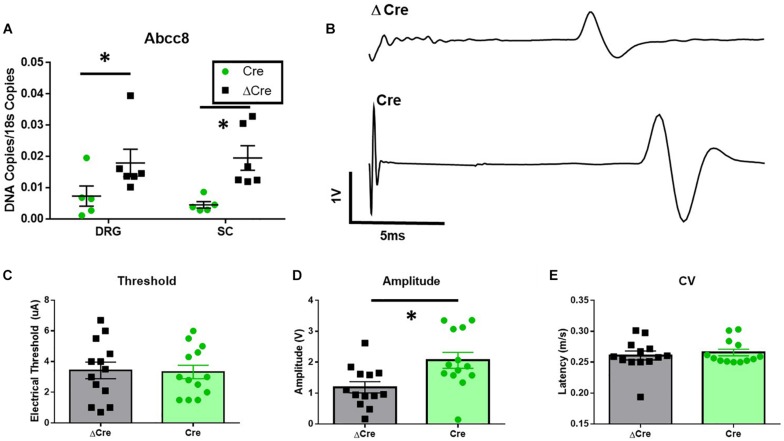
Increased sciatic nerve C-CAP amplitude following conditional deletion of SUR1 in mice. **(A)** Quantitative PCR data indicating the number of copies of *Abcc8* (protein: SUR1) are decreased in AAV9-hSyn-Cre mice compared to AAV9- hSyn-ΔCre mice (DRGs: unpaired *t*-test, *p* = 0.049; SC: unpaired *t*-test, *p* = 0.008, *n* = 6/group). **(B)** Example of sciatic nerve C-fiber CAP recording from AAV9-hSyn-ΔCre (top) and AAV9-hSyn-Cre (bottom) mice 8 weeks after inoculation. **(C)** No significant threshold differences between AAV9-hSyn-Cre and AAV9-hSyn- ΔCre mice. **(D)** Peak amplitude was significantly increased in AAV9-hSyn-Cre compared to AAV9-hSyn- ΔCre mice (unpaired *t*-test, *p* = 0.01, *n* = 13/group). **(E)** No significant differences in conduction velocity (CV) between AAV9-hSyn-Cre and AAV9-hSyn-ΔCre mice. Asterisk indicates statistical significance (*p* < 0.05).

To confirm loss of mRNA expression of *Abcc8*, qPCR experiments were performed on spinal cord and DRG tissues after completion of behavioral testing. *Abcc8* expression was significantly reduced in AAV9-hSyn-Cre compared to AAV9-hSyn-ΔCre inoculated mice, while other K_ATP_ channel subunits were largely unchanged (Figure 5A and data not shown, unpaired *t*-tests, *p* < 0.05). We hypothesized the increased mechanosensitivity could be due to the increased excitability of nociceptors innervating the periphery. The properties of C-fiber CAPs from AAV9-hSyn-ΔCre and AAV9-hSyn-Cre sciatic nerves (Figure 5B) were largely similar. In fact, no significant threshold differences or conduction velocity differences were found between sciatic nerves of AAV9-hSyn-Cre and AAV9-hSyn- ΔCre mice (Figures 5C,E), yet the peak amplitude was significantly increased in AAV9-hSyn-Cre compared to AAV9-hSyn- ΔCre mice (Figure 5D, unpaired *t*-test, *p* = 0.01).

### Mice With shRNA and Conditional Knockdown of SUR1-Subtype K_ATP_ Channels Have Decreased Morphine Antinociception, and Potentiated Morphine Tolerance and Withdrawal

**FIGURE 6:**
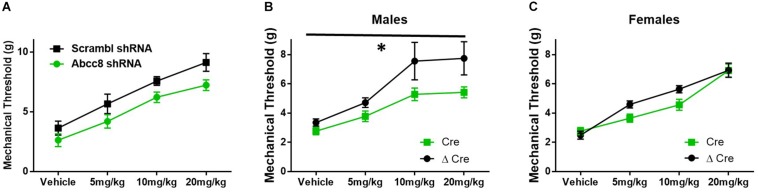
Morphine efficacy is decreased after *Abcc8* shRNA or conditional deletion of SUR1 in the spinal cord. Delivery of AAV9 viruses was performed via intrathecal injection. **(A)** Morphine antinociception is significantly decreased 4 weeks after AAV9-shRNA-Abcc8 versus a control (scramble) shRNA administration [repeated measures ANOVA, *F*(1,8) = 8.629, *p* = 0.018, *n* = 5/group]. **(B)** Mechanical paw withdrawal thresholds after morphine administration were significantly decreased 4 weeks after AAV9-hSyn-Cre virus compared to control virus treatment in male SUR1 cKO mice [AAV9-hSyn-ΔCre, repeated measures ANOVA, *F*(1,20) = 8.428, *p* = 0.009, *n* = 11–12/group]. **(C)** Mechanical paw withdrawals between viral groups were unchanged in female SUR1 cKO mice. Asterisk indicates statistical significance (*p* < 0.05).

Mice lacking SUR1 globally have attenuated responses to morphine (Figure 3), so morphine antinociception was similarly tested in shRNA and SUR1 flox mice. Morphine antinociception was significantly decreased after AAV9-shRNA-Abcc8 versus AAV9-shRNA-scrambl administration [Figure 6A, repeated measures ANOVA, *F*(1,8) = 8.6, *p* = 0.018, *n* = 5/group]. Mechanical paw withdrawal thresholds after morphine administration were significantly decreased after AAV9-hSyn-Cre compared to AAV9-hSyn-ΔCre treatment in male SUR1 flox mice [Figure 6B, repeated measures ANOVA, *F*(1,20) = 8.4, *p* = 0.009), *n* = 11–12/group]. Unexpectedly, female SUR1 flox mice did not display a markedly decreased mechanical paw withdrawal thresholds compared to the control vector group [Figure 6C, repeated measures ANOVA, *F*(1,23) = 2.45, *p* = 0.13, *n* = 12–15/group]. Overall, the analgesic efficacy of morphine is greatly attenuated in mice with downregulated SUR1-subtype K_ATP_ channels in the lumbar spinal cord and DRG.

**FIGURE 7:**
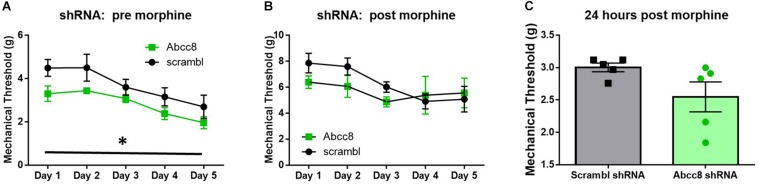
Development of hyperalgesia following repeated morphine administration is potentiated after *Abcc8* shRNA treatment in mice. Morphine tolerance was established by twice daily injections of 15 mg/kg (s.c.) morphine. **(A)** Five to six weeks after intrathecal injection of AAV9-shRNA-Abcc8 at the lumbar spine level, baseline mechanical paw withdrawal thresholds (pre morphine) were significantly lower than AAV9-shRNA-scramble treated mice [repeated measures ANOVA, *F*(1,8) = 18.86, *p* = 0.003, *n* = 5/group]. **(B)** Mechanical paw withdrawal responses after morphine administration (15 mg/kg, s.c., 30 min) were not significantly lower than AAV9-shRNA-scramble treated mice. **(C)** Mechanical thresholds were not significantly altered between AAV9-shRNA groups 24 h post morphine administration. Asterisk indicates statistical significance (*p* < 0.05).

We predicted localized knockdown of SUR1 would potentiate the development of morphine tolerance and increase mechanical hypersensitivity during withdrawal. Five-to-six weeks after intrathecal injection of AAV9-shRNA-Abcc8 at the lumbar spine level, baseline mechanical paw withdrawal thresholds before morphine injection (pre- morphine) were significantly lower than AAV9-shRNA-scramble treated mice [Figure 7A, repeated measures ANOVA, *F*(1,8) = 18.9, *p* = 0.003, *n* = 5/group]. Thirty minutes after morphine administration (post morphine) mechanical paw withdrawal responses were not significantly lower than AAV9-shRNA-scramble treated mice [Figure 7B, repeated measures ANOVA, *F*(1,8) = 1.6, *p* = 0.24]. On day six, 24-h post-completion of the chronic morphine administration, the mechanical threshold data were not significantly altered between AAV9-shRNA groups (Figure 7C, unpaired *t*-test, *p* = 0.095). It appears after knockdown of *Abcc8* (protein: SUR1), the development of morphine tolerance is not greatly potentiated, but the development of opioid-induced hypersensitivity (pre-morphine) is significantly increased.

**FIGURE 8:**
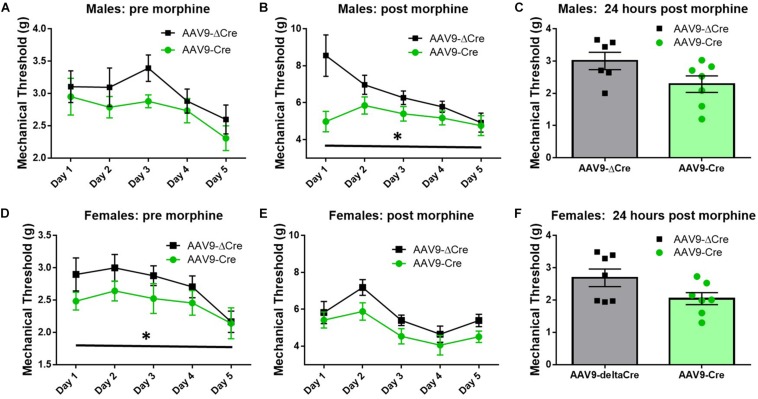
Morphine induced hyperalgesia and morphine tolerance are potentiated in mice conditionally lacking SUR1. Morphine tolerance was established by twice daily injections of 15 mg/kg morphine (s.c., *n* = 11–15/group). **(A)** Five-to-six weeks after intrathecal injection of AAV9-hSyn-Cre at the lumbar spine level, baseline mechanical paw withdrawal thresholds (pre morphine) were not significantly lower compared to AAV9- hSyn-ΔCre control mice. **(B)** Mechanical paw withdrawal responses after morphine administration (30 min) were significantly lower in AAV9-hSyn-Cre male mice than AAV9- hSyn-ΔCre control mice [repeated measures ANOVA, *F*(1,20) = 9.85, *p* = 0.005]. **(C)** Mechanical thresholds were not significantly altered between virus treated male mice 24 h post morphine administration. **(D)** Mechanical paw withdrawal responses before morphine administration were significantly lower in AAV9-hSyn-Cre female mice than AAV9- hSyn-ΔCre control mice [repeated measures ANOVA, *F*(1,23) = 3.364, *p* = 0.047]. **(E)** Mechanical paw withdrawal responses after morphine administration (15 mg/kg, sc, 30 min) were significantly lower in AAV9-hSyn-Cre female mice than AAV9- hSyn-ΔCre control mice [repeated measures ANOVA, *F*(1,23) = 5.75, *p* = 0.025]. **(F)** Mechanical thresholds were not significantly altered between virus treated female mice 24 h post morphine administration. Asterisk indicates statistical significance (*p* < 0.05).

Similar morphine tolerance and withdrawal data were also collected in AAV9-hSyn-ΔCre and AAV9-hSyn-Cre inoculated SUR1 flox mice. Five-to-six weeks after intrathecal injection of AAV9-hSyn-Cre in male flox mice, the baseline mechanical paw withdrawal thresholds (pre morphine) were not significantly lower compared to control male mice [Figure 8A, versus AAV9-hSyn-ΔCre, *F*(1,20) = 3.2, *p* = 0.09]. However, the mechanical paw withdrawal responses after morphine administration (post-morphine) were significantly lower in AAV9-hSyn-Cre male mice than AAV9- hSyn-ΔCre control mice [Figure 8B, repeated measures ANOVA, *F*(1,20) = 9.9, *p* = 0.005]. These data were similar, but not identical in female SUR1 flox mice. For example, mechanical paw withdrawal responses before morphine administration were significantly lower in AAV9-hSyn-Cre female mice than AAV9- hSyn-ΔCre control mice [Figure 8D, repeated measures ANOVA, *F*(1,23) = 3.4, *p* = 0.047]. The mechanical paw withdrawal responses after morphine administration were also significantly lower in AAV9-hSyn-Cre female mice than AAV9- hSyn-ΔCre control mice [Figure 8E, repeated measures ANOVA, *F*(1,23) = 5.8, *p* = 0.025]. Mechanical thresholds were not significantly altered between virus treated male or female mice ∼18 h post morphine administration (Figures 8C,F, unpaired *t*-test, *p* = 0.11 and 0.074, respectively).

### Local Delivery of K_ATP_ Channel Agonists Prevent Tolerance and Improve Mechanical Hypersensitivity During Morphine Withdrawal

**FIGURE 9:**
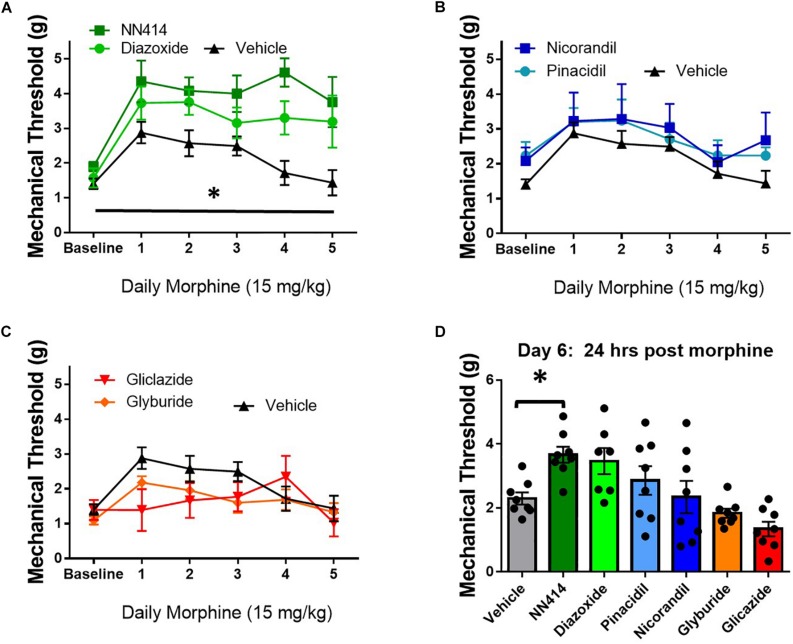
Local delivery of K_ATP_ channel agonists reduces morphine tolerance in SNL mice. Morphine tolerance was developed by twice daily administration of 15 mg/kg (s.c.) morphine. Mechanical thresholds were tested 30 min post morphine injection and ∼20 min post K_ATP_-targeting drug application (10 μL, intraplantar, 100 μM, *n* = 7–8/group). **(A)** The Kir6.2/SUR1 agonist, NN414, and the SUR1 agonist diazoxide attenuated tolerance compared to vehicle treatment [repeated measures ANOVA, *F*(6,48) = 7.741, *p* < 0.001, CI _NN414_ = –2.77 to –0.6266, CI_Diazoxide_ = –2.146 to –0.075]. **(B)** The SUR2 targeting agonists, pinacidil and nicorandil or the SUR1 and SUR2 targeting antagonists **(C)** did not attenuate morphine tolerance. **(D)** Mechanical thresholds were significantly higher in mice administered NN414 [ANOVA, *F*(6,48) = 6.300, *p* < 0.001, CI_NN414_ = –2.614 to –0.1216] in morphine withdrawn animals. Asterisk indicates statistical significance (*p* < 0.05).

K_ATP_ channel activity is positively correlated with opioid antinociception and opioid currents in *in vitro* studies (Welch and Dunlow, 1993; Cunha et al., 2010). Since decreasing K_ATP_ channel activity appeared to potentiate opioid tolerance and opioid-induced hyperalgesia (OIH), the local delivery of K_ATP_ channel agonists was tested to improve morphine antinociception during tolerance and withdrawal in SNL animals. Morphine tolerance was established by twice daily administration of 15 mg/kg morphine s.c. for 5 days. Mechanical thresholds on the ipsilateral paw were tested 30 min post morphine injection and ∼20 min post K_ATP_-targeting drug administration through intraplantar injection (10 μL, intraplantar, 100 μM, *n* = 7–8/group). Intraplantar delivery of the Kir6.2/SUR1 agonist, NN414, and the SUR1 agonist diazoxide attenuated tolerance compared to vehicle treatment [Figure 9A, repeated measures ANOVA, Dunnett *post hoc*, *F*(6,48) = 7.7, *p* < 0.001, CI_*NN*414_ = −2.77 to −0.6266, CI_*Diazoxide*_ = −2.146 to −0.075]. Intraplantar delivery of the SUR2 targeting agonists, pinacidil and nicorandil did not significantly attenuate tolerance (Figure 9B). It was predicted that intraplantar delivery of the SUR1 and SUR2 targeting antagonists, gliclazide and glyburide, would escalate morphine tolerance. Although the mechanical paw withdrawal thresholds were lower than vehicle on days 1–3, no significant differences were found using the K_ATP_ channel antagonists (Figure 9C). On day 6, ∼18 h after the last morphine injection, mechanical paw withdrawal thresholds were taken 20 min post vehicle or K_ATP_ channel agonist/antagonist intraplantar injection. Mechanical thresholds were significantly higher in morphine withdrawn mice administered NN414 [Figure 9D, ANOVA, Dunnett *post hoc*, *F*(6,48) = 6.3, *p* < 0.001, CI_NN414_ = −2.614 to −0.1216]. Neither the SUR2-subtype targeting agonists, nor the SUR1 or SUR2 antagonists had a significant effect on paw withdrawal thresholds compared to vehicle treated-morphine withdrawn mice. Data obtained from contralateral paws were not significantly different across groups (data not shown). Pharmacological targeting the peripheral nervous system with SUR1-subtype K_ATP_ channel agonists, appears to be beneficial to alleviate the development of morphine tolerance and withdrawal.

## Discussion

These results indicate K_ATP_ channel subunit expression and/or activity after chronic morphine exposure is dependent location within the nervous system. Genetic deletion of the SUR1-subunit in the lumbar spinal cord and DRG attenuated morphine antinociception and potentiated morphine tolerance and withdrawal, with some effects being sex dependent. Our pharmacology results indicate that introduction of agonists targeting the SUR1-subtype of K_ATP_ channels significantly inhibited the development of tolerance.

The results presented here show the expression of K_ATP_ channels during morphine tolerance is highly dependent on whether underlying nerve injury was present or absent in mice. In this study, the pore-forming subunit *Kcnj11* iso1 was significantly downregulated in DRG from MT + SNL mice, an finding not seen between ipsilateral and contralateral DRG of SNL mice in a previous study (Luu et al., 2019). Conversely, *Kcnj11* iso2 was previously found to be significantly downregulated in SNL animals (Luu et al., 2019), but *Kcnj11* iso1 downregulation was only found on the ipsilateral side of MT + SNL animals. *Kcnj8*, which is not reported to be expressed in the peripheral nervous system (Zoga et al., 2010; Wu et al., 2011), was significantly upregulated in MT + SNL animals. Similar trends between MT and MT + SNL mice were also seen in the regulatory Abcc subunits. *Abbc9* subunits were found to be consistently upregulated in MT + SNL mice, whereas *Abcc8* subunits were upregulated in the spinal cord, but downregulated in DRG. The data were presented as a change compared to saline treated animals after normalization for consistency. It is possible that our data transformation could have resulted in “basement” effect, where the data has a lower limit to the values we can collect. This could explain why some ratio changes were quite large, but failed to reach statistical significance.

In our previously published studies, *Kcnj11* and *Abcc8* subunits were significantly decreased in the peripheral nervous system of SNL mice, but not the spinal cord (Luu et al., 2019). We conclude that the addition of chronic morphine treatment can create significant changes in K_ATP_ channel expression the nervous system, with or without underlying chronic neuropathic pain. Potassium flux in SH-SY5Y cells or DRG from mice chronically treated with morphine was decreased compared to controls without morphine pre-treatment. Although these differences were small, we believe these results warrant further investigation on how the loss of K_ATP_ channel function, in addition to expression, contributes to morphine tolerance and withdrawal. In this study, whole spinal cord or DRG were used for FLIPR and RNA extraction used for mRNA expression. We did not sort neurons according to size, or by cellular markers etc., nor where they separated from satellite cells or other non-neuronal tissues. This is important to note as previous *in vitro* studies on DRG neurons indicate that K_ATP_ currents are not expressed in all neurons (Sarantopoulos et al., 2003; Chi et al., 2007) and different K_ATP_ channel subunits are also expressed in various cell types, including astrocytes (Wang et al., 2013), microglia (Ortega et al., 2012), and even vascular endothelial cells (Aziz et al., 2017). The changes in K_ATP_ subunit mRNA expression or cultured DRG potassium flux data reported after morphine tolerance, and especially after nerve injury, could be due to the aggregation of changes across multiple cell types, potentially in opposing directions depending on the cell and tissue location.

Earlier pharmacological studies have indicated K_ATP_ channel agonists could be used as a therapeutic during morphine tolerance (Seth et al., 2010; Cao et al., 2016). Our pharmacological data are in alignment with a previous study indicating the non-selective SUR1/SUR2 agonist cromakalim and the SUR1 agonist diazoxide could inhibit phenotypic indicators of morphine withdrawal in rodents including the jumping and fore-paw tremors (Robles et al., 1994; Thomzig et al., 2005). In our studies, the paw withdrawal thresholds on Day 6 after morphine tolerance was significantly attenuated by NN414, but not diazoxide. The EC_50_ for K_ATP_ channels composed of the SUR1/Kir6.2 subunits is ten times lower for NN414 compared to diazoxide (Martin et al., 2016). Although NN414 and diazoxide both attenuated morphine tolerance, the increased potency for the neuronal SUR1/Kir6.2 subtype might explain the enhanced analgesic efficacy of NN414 in our studies.

The idea that K_ATP_ channel activation is beneficial for opioid tolerance and withdrawal has been challenged in other studies (Khalilzadeh et al., 2008; Khanna et al., 2010; Singh et al., 2015). A recent study has implicated morphine-evoked TLR4-NLRP3 inflammasome-mediated neuroinflammation in microglia is important for morphine tolerance, and the K_ATP_ channel blocker glyburide (i.e., gliblenclamide) could inhibit this morphine-induced activation of the NLRP3 inflammasome (Qu et al., 2017). Our data indicate *Kcnj8* (protein: Kir6.1) and *Abcc9* (protein: Sur2) expression significantly increased in the spinal cord of MT + SNL mice. Kir6.1 has been shown to be the primary pore-forming subunit of K_ATP_ channels in astrocytes and in microglia (Thomzig et al., 2001; Zhou et al., 2001). SUR2 has been found in neurons, astrocytes, and occasionally microglia (Wu et al., 2011), whereas Kir6.2 and SUR1 are the principal subunits in neurons. Recently, compelling evidence show that glia cells, including both microglia and astrocytes, also play a pivotal role in chronic pain and morphine tolerance. The K_ATP_ channel opener cromakalim was found to reduce neuropathic pain partially via regulating gap junctions in astrocytes (Wu et al., 2011), suggesting K_ATP_ channel activity in non-neuronal cells can also contribute to acute analgesia. We suggest that Kir6.1/SUR2- glial K_ATP_ channels may normally act in synergy with neuronal Kir6.2/SUR1- K_ATP_ channels to produce antinociception with or without acute exposure to opioids, but this relationship can somehow be inversed during chronic exposure to opioids. This evidence is corroborated by our previous data indicating that SUR2 targeting agonists can alleviate neuropathic pain in mice (Luu et al., 2019), but this effect appears to be lost after chronic morphine exposure (Figure 9B). Future experiments utilizing viral vectors with glial promotors (e.g., GFAP or Iba1) targeting Kir6.1 and/or SUR2 could help to explain the current discourse regarding the role of K_ATP_ channels during chronic opioid exposure. The possibility that opioid analgesic effects can be separated from opioid tolerance and withdrawal via nervous system cell type is an exciting possibility corroborated by genetic studies in mice (Corder et al., 2017). Further studies are needed to examine K_ATP_ channel subunit expression and functional changes in discrete cell types and location(s) in order to achieve a more detailed picture of neuronal and non-neuronal components during chronic pain and chronic exposure to opioids.

K_ATP_ channels may play a major role in large diameter DRG neuron-mediated neuropathic pain (Zoga et al., 2010), and the loss of SUR1-subtype K_ATP_ channels in mice have been found to produce a small degree of mechanical hyperalgesia (Luu et al., 2019). Similar data were collected using mice lacking SUR1 in the lumbar spinal cord and DRG. After morphine administration, SUR1 KO mice had attenuated antinociception compared to SUR1 WT mice; an effect that was mirrored, to a lesser degree, in AAV9-shRNA and AAV9-hSyn-Cre virus treated animals. Alterations in sciatic nerve excitability were found after the loss of SUR1, due to intrathecal delivery of the AAV9-hSyn-Cre virus, demonstrating the consistency of behavioral results with this theory. Interestingly, there appeared to be a sex effect that was present in AAV9-hSyn-Cre virus treated animals when testing efficacy for morphine antinociception, before and after morphine during the tolerance studies, and even when assessing burrowing behaviors. K_ATP_ channel activity is known to be altered by estrogen and testosterone *in vitro* (Sakamoto and Kurokawa, 2019), however there are very little data regarding these effects *in vivo*, and almost no data from the nervous system. A small sex effect could be seen in the SUR1 Het and KO mice when testing for morphine antinociception, but this effect was not statistically significant. It is possible that a global knockout of SUR1 could result in compensatory up-or-down regulation of other channels/receptors to negate these sex effects seen when SUR1 is downregulated in adult animals. Further studies are needed to determine the effects of sex hormones on K_ATP_ channel activity in the nervous system before any therapeutics can be utilized in the clinic.

We could also speculate that a loss of K_ATP_ channels in the peripheral nervous system could ultimately affect second order neurons in the spinal cord, by altering mechanoreceptor input from large diameter neurons. In SUR1 KO animals, loss of morphine antinociception during mechanical paw withdrawal testing was greater than in thermal paw withdrawal testing. Almost all large nociceptors express μ-opioid receptor mRNA in very low amounts (Silbert et al., 2003). Instead, we suggest that opioids can reduce nociceptive signal transmission at central terminals of Aδ- and C-fiber synapses in the spinal cord (Heinke et al., 2011), which is lost after SUR1 knockdown. In AAV9-shRNA and AAV9-hSyn-Cre virus treated animals, a decrease in burrowing and rotarod activity was also observed in mice following conditional deletion of SUR1. These data are important as previous studies have indicated non-evoked measures of pain sensitivity, such as burrowing or rotarod, are beneficial to investigating phenotypic differences between animals of different chronic pain models or genetic modifications (Sheahan et al., 2017; Shepherd et al., 2018). Collectively, these data suggest that loss of SUR1-subtype K_ATP_ channels are important for pain signaling, and loss of SUR1 could promote mechanical hyperalgesia and increase spontaneous measures of pain.

The conditional deletion of SUR1 subunits in the spinal cord and DRG reduced the efficacy of morphine and potentiated tolerance in male, but not female mice. Conversely, OIH (pre-morphine) was significantly enhanced in female but not male mice. Mechanical paw withdrawal thresholds were similarly affected across AAV9-hSyn-Cre male and female mice, but burrowing behavior was affected significantly more in male versus female mice. These differences could be due to circulating hormones that can affect K_ATP_ channel activity. Testosterone has been found to increase K_ATP_ channel currents in smooth muscle cells (Han et al., 2008) and mitochondria (Er et al., 2004). Conversely, estrogen has been found to decrease K_ATP_ channel activity in beta cells (Soriano et al., 2009) but increase expression in cardiomyoctyes (Ranki et al., 2002), which is not reported in males (Ranki et al., 2001). Unfortunately, there is a lack of data on the hormonal modulation of K_ATP_ channel activity and expression in the nervous system that could help us to clarify exactly how biological sex influences ion channel, particularly potassium, flux during chronic pain and chronic opioid exposure. Interestingly, the global SUR1 KO mice have not been reported to have differences in mechanical or thermal sensitivity with regards to sex (Luu et al., 2019), even after acute morphine exposure (Figure 3).

Opening or closing K_ATP_ channels appears to affect morphine tolerance and/or withdrawal, but the exact molecular or cellular mechanisms are currently difficult to postulate. Chronic pain is closely associated with opioid tolerance, and both of these phenomena may occur due to similar changes in intracellular signaling pathways in in the peripheral nervous system and spinal cord (Chen et al., 2017; Araldi et al., 2018; Ferrari et al., 2019). Increased K_ATP_ channel activity may help to alleviate opioid resistance by decreasing hyperalgesic priming found in chronic pain states and/or chronic opioid administration. Stimulation of potassium channels due to opioid receptor activation causes neuronal hyperpolarization, which may inhibit voltage-gated calcium channels or other channels (Dembla et al., 2017). It is also possible K_ATP_ channel agonists mimic the effects of morphine on neuronal potassium currents and may act as a replacement for the lack of opioid-induced pro-analgesic intracellular signaling or compensate for hypertrophied pro-nociceptive signaling during morphine tolerance and withdrawal (Williams et al., 2001, 2013).

In addition to morphine (Ocaña et al., 1990), it has been suggested that K_ATP_ channels may mediate the analgesic effects of other drugs including pregabalin (Kweon et al., 2011), clonidine (Ocaña and Baeyens, 1993), U50,488H (kappa opioid agonist) (Ocaña and Baeyens, 1993), amytripyline (Hajhashemi and Amin, 2011) and (±)-8-hydroxy-2-(di-*n*-propyl-amino) tetralin (8-OH-DPAT, a 5-HT1_*A*_ agonist) (Robles et al., 1996) and JWH-015 (CB2 receptor agonist) (Reis et al., 2009). Interestingly, morphine withdrawal is inhibited by several of these same non-opioid drugs such as clonidine (Gossop, 1988), 5-HT agonists (Dzoljic et al., 1990), pregabalin (Hasanein and Shakeri, 2014), and cannabinoids (Vela et al., 1995). These data indicate that opening K_ATP_ channels may mediate a common pathway to promote hyperpolarization, and therefore analgesia, through many types of neurons. Activation of concurrent signaling pathways may help to explain the analgesic effect of K_ATP_ channel agonists, especially in inflammatory and neuropathic pain models (Du et al., 2011; Niu et al., 2011; Wu et al., 2011; Afify et al., 2013; Luu et al., 2019).

The study clearly indicated K_ATP_ channels play an important role in morphine signaling, and their modulation by either pharmacological or genetic intervention decreased opioid antinociception, and increased hypersensitivity during tolerance and withdrawal. We currently do not know if this channel is involved in any adverse effects of morphine such as respiratory depression, constipation, addiction, or dependence, but our present data utilizing *in vivo* and *in vitro* models provide a solid framework for pursuing further research into these areas. Future implementation of K_ATP_ channel pharmaceutics during opioid therapy may reduce the severity of one or more of these adverse effects and could be valuable in the clinical management of opioid tolerance, opioid induced hypersensitivity, and withdrawal.

## Data Availability Statement

The datasets generated for this study can be found in the Data Repository for University of Minnesota (DRUM) https://conservancy.umn.edu/discover.

## Ethics Statement

The animal study was reviewed and approved by the University of Minnesota Institutional Animal Care and Use Committee.

## Author Contributions

CF, KJ, TO, TJ, ES, AN, MM, AD, and AK performed the experiments and analyzed the data. AK finalized the figures, wrote the manuscript drafts, and conceived the studies. All authors reviewed and edited the manuscript.

## Conflict of Interest

The authors declare that the research was conducted in the absence of any commercial or financial relationships that could be construed as a potential conflict of interest.

## Footnotes

1 www.dapsys.net
